# Acute Anterior Poliomyelitis

**Published:** 1911-08

**Authors:** T. Jos. Dean


### ACUTE ANTERIR POLIOMYELITIS.

By T. Jos. Dean, M. D.

Infantile paralysis is no new disease, nor do I propose to advance any new thought in its pathology or treatment. Perhaps there is no disease with which the human race is afflicted that should deserve more consideration by the medical profession; and if I succeed in eliciting a free and comprehensive consideration by the members of this honorable body, thus keeping the question agitated until some one who may yet be unborn may discover its true exiology, the object and desire of this paper will be accomplished. For years, we have waited and watched for some scientist to discover the hidden secret by which the disease could be prevented or checked in its incipiency, and thus save the unfortunate child of the fate of a paralytic for life. We have looked in vain for the discovery, notwithstanding much advance has been made. However close we seem to it, the exact etiology of acute anterior poliomyelitis has not as yet been made clear. Since the majority of cases occur in summer, beginning with fever, diarrhoe and vomiting, there seems to be reason to believe that the bacterial infection in the intestine generates a toxemia which may be an etiological factor.

The fact that the disease appears in epidemic, points to the possibility of bacterial invasion. From the exhaustive research of Flexner and Lewis, of the Rockefeller Institute, the disease has been proven to be due to a virus which will pass through the smallest filters. They have kept the virus alive through more than twenty generations by injecting an emulsion from the brain and spinal cord of human beings who die from poliomyetitis into the brain of monkeys. This produces the typical symptoms with paralysis in the infected monkeys, and their brains and spinal cords are in turn capable of infecting other animals. They have also discovered an immunity in monkeys and human beings who recover from the disease, which gives an atoxic value to the blood serum. By using the blood serum of monkeys immunized against the disease they were able to prevent paralysis in monkeys injected with the active virus. Their conclusions are that if the quantity of virus injected into the brain eighteen to twenty hours before the serum treatment is begun, is not in excess of a given dose, the action of the virus can be prevented; and that the infection of the meninges from the nasal mucus can be prevented by serum injection. The attempts at immunizing horses in order that their serum might be used in practice, has been unsuccessful. It is claimed, beyond the reason of a doubt, that the virus of poliomyelitis is contained in the naso-pharybyed mucosa of inoculated monkeys, which would be a most likely method of spontaneous infection in the human. Recent investigation has shown that this virus exists and persists in an infectious state in this mucus menbrane for several months after the acute period of paralysis has passed, and for a much greater period than it survives in the central nervous system.

All attempts at growing the specific organism have proven failures, but it is our expectation and belief that in no distant date the organism will be grown in pure culture when its relationship to the disease can be proven.

Pathology: Acute anterior poliomyelitis is characterized by a sudden onset of fever, by an early and unusually extensive loss of power, followed by muscular atrophy and imperfect bone development, and sometimes, deformity. According to a recent contribution, the disease is characterized by a general toxermia affecting the heart, liver and kidneys, and the bymphoid tissues as in other specific infectious diseases. Holt's table shows that the greatest number of cases develop before the fifth year, and that the most frequent period being the second year.

Boys are more frequently affected than girls. The great proportion of cases occur in summer. Of Sinkler's cases, eighty percent began during the five warm months. This is decidedly against the theory that the disease results from the exposure to cold. Goldscheider believes that a condition of irritation is present in the walls of the blood vessels of the cord leading to their dilitation and to the proliferation of their endothelial elements. Later degenerative changes in the ganglion cells, as well as in the new fibres appearing in the vicinity of the altered blood vessels. The clinical data show this to be an invasion of bacteria, although it has not yet been proven. The inflammatory process is shown on transversa section of the cord that it is in the gray matter of the anterior horn. The brain stem and basal ganglie may be involved.

Ederma and destruction of the ganglion cells of the anterior horns are always found present, as is also an early degeneration of nerve fibres from the anterior roots.

The older idea that the destruction of the anterior horns is due to embolism and thrombosis of the vessels is only partly true. The picture is that of a specific infection, localized particularly in the anterior cornua, and often involving the adjacent white matter of the cord.

In the study of acute anterior poliomyelitis, three facts at once appear prominent: 1st. It is essentially a disease of early childhood. 2nd. It occurs most frequently in warm weather. 3rd. It is often epidemic.

If the older text-books on nervous and children's diseases are consulted, we small find a fourth prominent feature: namely, its relation to, or association with contagious diseases, especially measles. While these three features are all common, and, as a rule, hold good in the disease, none of them is essential to the diagnosis, as it occurs sporadically, at all seasons, and at all ages.

It is believed to be more common in America than any other country, and next in Scandinavia, although its racial and geographic distribution is rather extensive. In view of recent knowledge, the congenital idea of poliomyelitis can scarcely be true. Certainly, however, it does occur in very young infants, and cases have been reported in patients over seventy. The majority of adult cases occur before thirty.

Prodromal symptoms are rare in poliomyelitis, a sudden onset is one of its prominent characteristics. The invasion is almost always rapid, and often sudden. After a period of restlessness, sometimes lasting only a few days, the child is found to be paralized. The child may awake in the morning paralized after apparently a restless night. Usually there is a short febrile period before the paralysis. The temperature is usually moderate, from 100 to 102, then, it may rise to 103 or 104. Vomiting and delirium are common in this stage. If convulsions are present, they are not severe. Pain and other sensory symptoms are not prominent. The absence of these symptoms is one of the characteristics of poliomyelitis. This is readily appreciated when we consider its morbid anatomy. The clinical picture is simply one of an acute infection, and practically the same as may be found in other infections. This stage may last from a few hours to a week—usually, it is less than a day.. Then, the dominant symptoms, the motor paralysis appears.

The paralysis is oftenest paraplegia. It may be monoplegia, one arm, or more frequently, one leg, various combinations of muscles may exist. According to Danna, the muscles of the eye, larynx and respiratory muscles always escape in infants. The rapid development of the paralysis is followed by a period of one to four weeks' duration, in which but little change is seen in the affected muscles. This is followed by spontaneous improvement, which, according to Gowers, begins in the muscles last effected, and reaching the limit in about three months. After this time, very little improvement is to be looked for. The paralyzed limb is flacid. Its reflexes disappear, and the temperature is lower than in the sound limb. There is never anaesthesia, and tenderness of the muscles is common. The atrophied muscles begin to lose their power to respond to forodism very early in the disease. Certainly muscles will improve, and preserve some degree of contractibility, although this is never normal, but improves later. The atrophy after reaching a certain stage ceases, and then, may improve slightly, even in permanently paralized limbs. Contracure of unopposed muscles cause deformity, and it is not uncommon to see curvatures of the spine and other deformities from this non-muscular development. The curvature of the spine is the result of the involvement of one of the lower extremities, which on account of the shortened condition allows the body to careen to one side; thus the curvature is produced.

The most frequent deformity resulting from this affection is tolipes varus—next is talipes varus, both these being associated with a certain amount of equimus. Sacks and Remak have noted an interesting fact that where all the muscles of a limb are not paralyzed, or only partly so, those functionally related, rather than those anatomically, are affected. Thus in the forearm, the supinator longus may escape when all the extensors of the forearm are involved. Where the paralysis occurs in the upper arm, the supinator longus is affected in connection with paralysis of the deltoid, biceps and bradrealis anticus. In the lower group, the tibialis anticus is paralized in connection with the quadriceps extensor. These muscles, we know, are supplied by different nerves, but they are all associated in the extensor movements of the limb.

When we stop to consider the pathology, distribution of lesions and functions of the parts involved, the paralysis and symptoms of a fully developed case of the disease is readily explained. It is an acute exudative inflammation of the anterior horns, chiefly of the spinal cord. This inflammation is not generally diffuse—at best, not equally so—certain groups of cells being chiefly affected. These cells become destroyed, more or less, and connective tissue takes their place. The anterior cornua contains not only the motor cells of the cord, but also the trophic cells. Therefore, there is not only motor paralysis corresponding to cell groups involved, but early strophy. This muscular atrophy is easily understood, since the nerve supply is cut off, exercise ceases, and the tropic cells are destroyed.

As to the diagnosis of poliomyelitis, the acute onset, the rapid wasting, the spontaneous improvement in certain groups of muscles, the absence of sensory symptoms, and finally the reaction of degeneration, all combined to constitute a type which it is difficult to confound with any other disease.

While it is difficult, or well-nigh impossible to make a diagnosis until the paralysis has taken place, the now generally recognized communicableness of the disease makes an early diagnosis extremely important. The microscope has been brought into play at this time, with some degree of satisfaction, but with all the laboratory methods, we are still unable to give a positive answer until the paralysis appears. There is nothing to particularly distinguish the stage of invasion from other acute infections. In the search for early symptoms emphasis has been placed on the great prostration of the patient which sometimes appears early, and apparently, out of proportion to the fever and other symptoms; but this is present in other infections. Some claim that there is great helplessness of the limbs before it is completely paralized, or function is lost; but this condition may be explained by the general prostration and lassitude of the little patient, and by the fact that paralysis may already be setting in.

After the paralysis is fully established, and its type can be determinated, there is usually little difficulty in reaching a conclusion—excepting cases in very young children, in whom only limited muscle groups are affected. In well developed cases, with a reasonably complete history, a diagnosis should be fairly certain. I have no doubt that some cases which have been reported as examples of poliomyelitis, terminating in complete recovery, were really cases of multiply neuritis. But "the acute onset, the rapid wasting, the spontaneousimprovement in certain groups of muscles, the absence of sensory symptoms, and finally the re-action of degenaration—all constitute a type which it is difficult to confound with any other disease."

As to the *prognosis,* it is difficult to state what will be the outcome of a case of this kind. It seems that the severity of the beginning of an attack is no guide as to its outcome. Some mild cases may leave permanent deformity, but, as a rule, in all cases some muscles remain parmanently paralized.

*Treatment*:— Unfortunately, the largest number of cases do not come under observation of the physician in the acute stage, or the true nature of the disease is overlooked until the paralysis has appeared. The indication, in the early stage, is to produce sweating by hot baths, and to produce counter irritation to the spine by the means of dry cups, the paguekin cautery, or an ice bag may be applied to the spine. At the very beginning, we should not overlook the patient's hygenic surroundings. A tepid sponge bath should be given the patient every day, and if convenient, the addition of some sea salt will improve the effects of the bath. This bath should be followed by massage and passive movements. Galvanism in a very gentle curren should be used with due respect to the child's feelings, but strong enough to produce muscular contractions To these measures should be added, if there is much fever, antipyretics—such as phenacetine, or other coal-tar derivatives with the proper precautions in their use. There is much doubt expressed as to whether drugs have much curative effect upon the disease. Theoretically, ergot is indicated, but it is hard to say how much good can be attributed to its use. It can be given with Potassium Bromide. The use of

Sodium Salicilas seems rational, and it has been employed with some advantage in epidemics of the disease. Usually, at this stage, the disease is treatedsymptomatically.

After all acute symptoms have subsided, which is usually at the end of about three weeks, our most reliable therapeutic agents are electricity and massage, and exercise of the parts through various mechanical appliances appropriately devised by the orthopedic surgeon.

In the last, or chronic stage, the treatment is almost entirely a matter of orthopedic surgery, and in many cases, splendid results will follow this method of treatment, and the reverse is true if left entirely to nature.

*Discussion on the puaper of Dr. Dean on Acute Anterior Poliomyelitis—*

Dr. Langley—This paper is particularly interesting to me at this time, as I have just had under my care a patient with this disease. Dr. Dean has fully covered the ground in every way, and I thank him for the paper.

Dr. Baker—From the prevalence of this disease this summer, I find it a subject of great annoyance, especially as we have had from 15 to 20 cases in Montgomery. The question of infection and contagion calls for our careful attention, and Dr. Dean brings this matter out thoroughly. Of the cases we have had in Montgomery, none could be traced to another, but we do not feel authorized not to use strict quarantine, and I advise all physicians to quarantine where they have these cases. Some claim it is infectious, others that it is not.

Dr. Sanders—Of course I cannot discuss this disease from a clinical point of view, not having seen the disease for a good many years, as my work (State Health Officer) has not been ot a character to bring me in the sick room, except to make such diagnosis of such infectious diseases as, under the law, must be controlled. I must say, as the other gentlemen have said, it is a very important question as to whether this disease is communicable. Of course some diseases are contagious where the evidence of contagion has not been established, but it does seem where there are so many cases in the same City, there should be some method of finding the connection of the cases, or the mode of conveyance. We all see and know this disease is becoming more common than it has been, especially in the South, and it is a very vital matter for us to get all the information as to the communicability of the disease, and as to the organ through which the infection travels. As yet this disease has not been placed on the list in this State as one of those which can be controlled by law. At present there are fifteen diseases n this State under the law. I am very much interested in the subject, and am waiting for all the light the doctors can throw on the subject, and the State Board stands ready to deal with it as a contagious disease, when the doctors so decide.

Dr. P. M. Lightfoot—I had one case, a negro child, which I thought to be malarial fever, and so treated it for a few days, until I found the child had no use of its lower limbs, and some other symptoms of this disease, then I decided it must be poliomyelitis. As to communicability. Four weeks later a neighbor who had a negro man working for him, had a child who had this disease. We found that this negro man had been sitting up with this negro child during the acute stage of the disease, then would come the next morning to this neighbor's house to work for him during the day. This latter case, a child of one and one-half years old, developed fever and eruption, etc., and in a short time lost the use of its lower limbs also, and in this case you can readily see I felt the coming of the man directly from the negro house where the disease was, caused the infection in the house of the white man. As to the treatment, I am using nothing except what is recommended in our text books, except I am using iodine over the spinal cord.

I would like to ask the doctor if any of his cases have developed the eruption.

Dr. Dean, in closing—I do not believe I have any further remarks to add. As to the skin eruption, I do not know that I have observed this in any of my cases except where I thought it due to massage or the use of drugs. I thank the gentlemen for their discussion.

